# Supplementary material for: Development of 16 novel EST-SSR markers for species identification and cross-genus amplification in sambar, sika, and red deer
Source: PLoS One. 2022 Apr 1;17(4):e0265311. doi: 10.1371/journal.pone.0265311 (PMC8975116; doi:10.1371/journal.pone.0265311)
Supplement: Attachment — Submitted filename: Response to Reviewers.docx [file pone.0265311.s004.docx]

We would like to submit our manuscript entitled: “Development of 16 novel EST-SSR markers for species identification and cross-genus amplification in sambar, sika, and red deer”. The manuscript number is PONE-D-21-07853, and this is the resubmission of the manuscript PONE-D-20-36516. After the previous rejection, we have reframed the manuscript and amend the content according to the comments. We appreciate the suggestions the reviewers provide. Together, they have helped improve the clarity and accuracy of our manuscript, increasing its readability and concentrating on its main goal. Below are reviewers' comments and our responses:

1. Reviewer1:

The objective behind annotation of transcriptome is missing in the introduction. Overall, I recommend rewriting the introduction, discussion and conclusion for better readability.

Reply:

We rewrite the manuscript and remove some transcriptomic data. Hence, our manuscript will be more focused on the detection of cross-species amplification and identification of deers.

2. Reviewer1:

The results need to be presented better- instead a long tables, a figure summarizing the tables would be better.

Reply:

We replace the previous tables (Table 2-4) with new tables (Table 2 and 3). In table 2, we combine each marker information of sambar, sika, and red deer in table 2. Second, we summarize the information of high polymorphic markers across three deer populations.

3. Reviewer1:

Introduction: The second objective of the study “…and identify the genes involved in regulation of antler growth” is suddenly introduced in the results. Please take care of the structure. Moreover, the second objective to identify genes involved in the regulation of the antler growth perhaps needs better study design and sampling. Please justify, what analyses helped in identification of genes involved in antler growth.

Reply:

To concentrate on the development of markers, we remove the description of identifying antler-growth-related genes.

4. Method: (i) Also, are the hybrids easily detectable morphologically? I ask this to be certain that the two hybrid deer samples are indeed hybrid. I would also recommend increasing the sample size of the hybrid deer (if possible).

Reply:

We review the studies of the morphology of hybrid at line 80-87. The ancestry of two hybrid were confirmed according to their owner and their genetics were examined by zfy and mitochondrial DNA at line 132.

5. Reviewer1:

(ii) A pair-wise relatedness estimated to justify that the tested samples did not include only closely related individuals (siblings, parent-offspring pair).

Reply:

The red deer and sambar sample were collected from diverse resource (different farms), hence may reduce the concern of only closely related individuals. The Formosan sika deer were collected from a restoration population (description at line 348-351). Hence, the inbreed genetic structure may be inevitable.

6. Reviewer1:

Result: EST-SSR developed for Sambar (tested for 45 individuals, 55 primers)- 52 amplified sika and 49 amplified red deer. 11 primers were polymorphic for 30 sika deer and 17 primers were polymorphic for 15 red deer samples. Loci 34 and 44 heterologous for sika deer. Loci 21, 9 and 17 were polymorphic for 3 species- hence can be used for species identification. But, finally 24 EST-SSR were selected/tested for species id (using Fst statitics). It is hard for the reader to keep track of all these numbers. So, I recommend a flowchart for microsat. discovery.

Reply:

We summarize the process with a flowchart Figure 1. Also, we remove complicate description of the marker we used in different deer population. Instead, we focused on markers which showed good transferability across three deer in Table 3.

7. Reviewer1:

Discussion should include other papers that have used similar approach for cross-species identification.

Reply:

We review studies of SSR transferability at line 334-345. Moreover, we propose that this paper is the first study discussing the transferability of EST-SSRs in deer.

8. Reviewer1:

Conclusion needs to be completely rewritten.

Reply:

We rewrite the conclusion and focus on the newly-established marker system.

9. Reviewer1:

Is sympatry the reason for hybridisation of Sika deers and red deer or the hybridisation between these two species is only for antler?

Reply:

In Scotland, sympatry has led to hybridization of sika and red, which sika had been introduced due to the anthropogenic translocation. However, in deer farms, farmers mate sika and red for better antler production or mate red and wapiti for better venison production.

10. Reviewer1:

Line 12-14: Highlights the need for conservation of deer and then develops genetic markers to facilitate deer farming. The line about the need for conservation is not necessary in this manuscript.

Reply:

We emphasize the fear of anthropogenic translocation and the resulted problems of hybridization at line 20-22 in abstract and at line 46-54 in Introduction. We think the conservation issue is also an important target of our marker system.

11. Reviewer1:

Line 42: Please mention how many species within the family.

Reply:

We mention the number of deer species at line 47.

12. Reviewer1:

Line 86-88: So the SNPs developed so far shows polymorphism within species but not between species?

Reply:

We do not aim to discuss whether SNPs show lower polymorphism between species. Here we aim to discuss the weakness of current cross-species markers in deer. Few markers, including SNPs and SSRs, have been tested for the cross-genus amplification in deer.

13. Reviewer1:

Line 86-88: A comparison of performance of published SNPs (mentioned here) with the SSR marker developed in this study would be a better justification. A long justification for the use of microsatellite marker over SNP is provided. But, one of the main disadvantages of microsatellite markers are that they are expensive (all samples are usually tested in triplicates, scoring the microsatellite is prone to human error, etc.).

Reply:

Currently no published genetic analysis of our sampling deer (farmed red deer, Formosan sambar, and sika) based on these SNPs, which hampered the comparison.

About the advantages of SSRs, to our knowledge, the cost of sequencing (for SNP genotyping) is higher than capillary electrophoresis (for SSR genotyping). Hence, we propose using SSRs for a lower cost.

14. Reviewer1:

Line 89 – 102 compares the efficiency of SNPs and microsatellite in the context of species id, inbreeding, population structure, etc and ends with a very uninformative line “Microsatellites have a wide range of other applications, and for some of these they have been found to be more suitable than SNP”. The entire comparison can be moved to discussion and made it more specific to the currently developed microsatellite marker.

Reply:

We move the passage in discussion at line 323-334.

15. Reviewer1:

Line 131 – 139: These lines looks like method, should be moved to method section. The last paragraph of the introduction should describe the objectives of the study.

Reply:

We rewrite the paragraph and describe the objectives of this study.

16. Reviewer1:

Method line 142 – 155: Were all these samples (red deer n=46; sika-red hybrid n=2; sambar n=45, 1 sambar antler tip) pooled for transcriptome? First two paragraph describes the sample size but fails to convey how many samples were used to develop the marker and how many samples were used to test the marker. Please make this clear.

Reply:

We describe the genotyping sample collection at passage 1 and transcriptome sample at passage 2 to avoid confusion.

17. Reviewer1:

Move table 1 to supplementary.

Reply:

The main goal of this paper is to develop EST-SSRs. Therefore, we think the primer information is important to this paper and we want to keep the table in the manuscript.

18. Reviewer1:

Line 223-227: SSR validation: Rather than validating using clones, I think it would be ideal to test it on samples collected from wild. What is the purpose of cloning while developing microsatellite marker, please explain?

Reply:

We validate all alleles for two reasons. 1. The result sequence of De novo assembly may differ from the genomic DNA, hence we want to make sure the amplicon is our target sequence, which includes SSR sequence. 2. We want to make sure whether cross-species amplification would produce false amplicons.

19. Reviewer1:

Line 235 – 236: “To acquire EST sequences for SSR mining and to identify the genes involved in regulation of antler growth,…..”

Line 240-241: “Then, A de novo assembly pipeline was applied to these reads to generate a reference transcriptome.”

Reply:

To focus on marker development, we shorten the passage of de novo assembly and remove the description of growth-related genes.

20. Reviewer1:

Line 320-321: cross amplified in how many sika deer samples and red deer samples?

Reply:

The sampling number is shown in Table 2.

21. Reviewer1:

Line 326- 327: Did these 11 and 17 loci show polymorphism specific to species that were tested or were the polymorphism only within species? This is important to know where that same microsatellite can be used cross species or not. Right now, it is not clear.

Table 2,3 and 4 should be combined as a figure which is easy to understand. Some idea to do so: something like sample name in the y-axis and marker name/loci in the x-axis and filled circle to indicate the sample worked for that locus/marker.

Reply:

We rewrite this paragraph and remove the description of detailed polymorphic marker information within a single species. Instead, we summarize the mean value and combined value of polymorphic marker information in table 2.

22. Reviewer1:

Table 5: How many markers were used for the Fst. Please mention in the caption.

Reply:

We add the description in the table 4 caption.

23. Reviewer1:

Figure 3: From this figure it looks like only 2 samples of hybrid were tested. But from what I understand the objective of the paper is to develop markers which can identify hybrids. So, I recommend testing the developed markers on more hybrids samples and different hybrid types. It is not clear if the authors are interested on markers to detect hybrids of sika-red deer, red deer-sambar, sambar-sika. Or at least provide justification on why only markers specific for identifying red deer-sika hybrids was targeted.

Reply:

Our goal is to develop polymorphic EST-SSRs with good transferability. Hence, we focus on confirming the clear genotyping result and high polymorphism of markers. In Taiwan, hybridization is less practiced since farmers majorly keep sambar for antler production. Hence, only a few hybrids are accessible for preliminary testing. However, hybrids mating is common in China and New Zealand, and deer products are exported to other countries. We think these markers can help to resolve the identification of hybridization and fraud products worldwide in the future.

24. Reviewer1:

Line 452: “In this research, our focus was on exploring genes whose function contributes to the growth of antler velvet.” This objective is not mentioned anywhere else except the discussion.

Line 463-464:” These genes were both annotated in sika deer and sambar antler transcriptome, indicating their prevalence and importance in antler growth.” Since detection of gene in transcriptome does not entirely indicate their role for antler growth. This line should be rewritten or discussed in comparison to published literature that have identified genes relevant for antler development.

Line 464-465: “On the other hand, we were able to predict potential DEGs in sambar based on sika deer databases.” What is the relevance of this sentence?

Reply:

We remove the description of genes related to antler growth.

25. Reviewer1:

Line 471-473: “Cross-species transferability of these 55 sambar derived EST-SSR markers in sika deer (Cervus nippon) and red deer (Cervus elaphus) were 52 (94.5%) and 49 (89.1%), respectively….. We detected polymorphism by genotyping each species respectively, and the results showed that 21 loci (46.7%) in 45 sambar, 9 loci (18.4%) in 30 sika deer, and 17 loci (34.7%) in 15 red deer exhibited polymorphism.” What is the use of 55 markers showing cross species transferability when only 21, 9 and 17 show polymorphism? First line on 55 marker cross species transferability is not useful and can be removed.

Reply:

We rewrite the passage and focus on the success rate of the cross-species amplification of all 55 primer pairs. Moreover, we add the discussion of the cross-amplification in different taxa. These discussions are mean to support that our EST-SSRs features good transferability, and we are the first research comparing the genotyping transferability across deer species.

26. Reviewer1:

Line 481-482: Comment on inbreeding without information on spatial extent is not ideal.

Reply:

We remove the discussion about inbreeding.

27. Reviewer2:

The study entitled "De novo characterization of the sambar antler velvet transcriptome and EST-SSR development for cross-genus species-identification" is an interesting study in the face of increased threat of extinction of deer species. However, this study fails to define its research hypothesis clearly and tends to be verbose and confusing. The transcriptome analysis followed with SSR marker development and validation does not substantiate its primary objective of discriminating the source species of deer products and hybrids. The results produced in the study are also not discussed comprehensively. In line, the study does not comes up with a firm conclusion.

Reply:

We reframe the structure of the manuscript and concentrate on the issue of marker development. Moreover, we add discussion of SSRs transferability in other animal taxa and the advantages of EST-SSRs. We think our revision can corroborate our conclusion: the newly developed EST-SSRs are suitable for species identification and phylogenetic analyses across deer genera.

28. Reviewer:

Line 68: Please check sequencing spelling

Line 120: do can be removed.

Line 127: Check the spelling of sequence

Line 191-192: “To screen for loci with high polymorphism, PCR was applied to 45 sambar deer.” Rewrite as – “To screen for polymorphism, markers were tested on 45 sambar deer samples collected from zoo (?). ”

Line 301: “Statistical analysis of marker garnered data is shown in Table 2.” Please check this line.

Line 477: A sentence does not end with is. Please reframe

Line 488-492: The writing can be made better by rearranging the sentences. Conclusion: “In sum..” to be replaced as “In summary… ” Should be rewritten.

Reply:

We rewrite the sentence and ask for professional English editing to amend the manuscript.
